# Factors Associated with Mortality in Severe Acute Cholangitis in a Moroccan Intensive Care Unit: A Retrospective Analysis of 140 Cases

**DOI:** 10.1155/2021/4583493

**Published:** 2021-01-27

**Authors:** Soumaya Touzani, Abderrahim El Bouazzaoui, Fatima Bouyarmane, Kaoutar Faraj, Nawfal Houari, Brahim Boukatta, Nabil Kanjaa

**Affiliations:** Anesthesiology and Intensive Care Department A4, Hassan II University Hospital, Sidi Mohammed Ben Abdellah University, Fez, Morocco

## Abstract

**Background:**

Severe acute cholangitis is a life-threatening biliary infection, leading to organ dysfunction, septic shock, and naturally death. Mortality has dropped significantly in the past years through improving resuscitation and biliary drainage techniques. The aim of our study is to analyze our daily practice and the factors associated with mortality.

**Methods:**

A retrospective study including severe acute cholangitis patients admitted to our unit from January 2009 to December 2018. Variables analyzed (univariate then multivariate analysis) were age, sex, history, origin, evolution time, bilirubin, etiology, organ dysfunction, qSOFA, SOFA, TOKYO, biliary drainage timing and technique, shock, antibiotherapy, and resuscitation.

**Results:**

140 patients were included in this study. Average age was 61. Sex ratio M/F was 0.59. Lithiasis etiology was dominant (69%). SOFA average score upon admission was 8. Ceftriaxone + metronidazole was the empirical antibiotic used in 87%. Average time to biliary drainage was 1.58 ± 0.89 days. Endoscopic unblocking was the technique used in 76%. Mean duration of ICU stay was 6 days. Mortality rate was 28%. Statistically significant factors for mortality (*p* < 0.05) were history of taking anticoagulant treatment, use of catecholamines and mechanical ventilation during ICU stay, and delay in consultation and administration of antibiotic therapy.

**Conclusions:**

Early recognition, antibiotics, resuscitation, and minimally invasive biliary drainage have improved patient outcomes although there is still progress to be made. Moreover, as multiple organ failure is often associated with mortality in severe acute cholangitis, predictive risk factors of organ failure should be more investigated.

## 1. Introduction

Acute cholangitis is a bacterial infection of the biliary tract following cholestasis mainly caused by lithiasic or tumoral biliary obstruction. The classic pain-fever-jaundice symptoms, representing the Charcot triad, were first described in 1877 [1]. The severity of cholangitis is due to infection dissemination with risk of septic shock and organ failure. “The Reynolds pentad,” described in 1958 as Charcot triad-shock-confusion, was associated with high mortality in the absence of adequate treatment [2]. Early diagnosis and recognition of severe presentations are therefore a challenging task for every clinician in order to initiate an early and appropriate therapeutic management. This management is continuously progressing in terms of resuscitation, antibiotics, surgical techniques, and less invasive techniques such as interventional endoscopy and radiology. It therefore requires multidisciplinarity (anesthetists, intensivists, hepatobiliary endoscopists, surgeons, radiologists, and microbiologists) and the implementation of standardized protocols. The recommendations of “the Tokyo Guidelines Working Group” are regularly updated from 2007 to 2018 [1, 3–5]. Resuscitation, antibiotics, and biliary drainage as an early intervention approach are absolutely essential for survival in severe acute cholangitis. Mortality has certainly decreased from 50% to 10-30% over the past 30 years, but it remains high, and several questions still unanswered regarding the application of these recommendations on a local level. Furthermore, other factors may influence the prognosis. Recent studies consider admission to intensive care as a predictor of mortality [6]. Our aim in this study was to identify the factors associated to mortality in severe acute cholangitis in our context.

## 2. Materials and Methods

### 2.1. Study Design and Setting

In this retrospective observational monocentric study, we evaluated patients of age ≥ 16 years with severe acute cholangitis admitted to our Intensive Care Unit (ICU) between January 2009 and December 2018. The diagnosis of acute cholangitis was based on clinical-biological and radiological criteria according to the Tokyo guidelines. The severity was assessed by the presence of at least one organ failure and/or an unbalanced comorbidity. The criteria for diagnosis and severity are presented in Tables 1 and 2. Patients with incomplete and/or nonexploitable records were excluded from the analysis. The study setting was a 14-bed medico-surgical ICU in a tertiary university hospital in Morocco (ICU A4–Hassan II University Hospital of Fez). The study was approved by the institutional review board (Comité d'Ethique Hospitalo-Universitaire de Fès) with a waiver of informed consent.

### 2.2. Data Collection

Study data were collected retrospectively from both paper charts and electronic medical records of patients using HOSIX electronic data capture tools hosted at Hassan II University Hospital of Fez. Variables collected included demographic information, diagnostic parameters, the Sequential Organ Failure Assessment (SOFA) and quick-SOFA scores, and TOKYO grading upon ICU admission, patient comorbidities, therapeutics, and evolution.

### 2.3. Statistical Analysis

The statistical analysis of the parameters was performed using the SPSS 20 software in the epidemiology laboratory of the Faculty of Medicine and Pharmacy of Fez. Factors associated with mortality were analyzed using univariate and multivariate analysis. Descriptive statistics were used to summarize baseline patient characteristics. The results were expressed in numbers and percentages for the qualitative variables and in means ± standard deviations (SD) for the quantitative variables. Comparison of the quantitative and qualitative variables was based, respectively, on the Student's *t*-test and the chi-2 test (*χ*2) through univariate analysis. Both *p* values and odds ratio (OR) with corresponding 95% confidence interval (CI) were reported for qualitative (categorical) variables while only *p* values were presented for quantitative (continuous) variables. Multivariable logistic regression was used to identify variables associated with ICU mortality as the outcome variable of interest. Multiple logistic regression models were fitted by regressing mortality status on multiple clinical variables. Considering the large number of clinical variables included in the study, we chose a stepwise regression approach using backward elimination. Order of elimination was based on clinical relevance and statistical strength. The statistical significance threshold was determined at *p* = 0.05. Results of the multivariate analysis were shown as odds ratio (OR) and corresponding 95% confidence interval (CI). Most of the significant variables at the univariate analysis were entered in the multivariate analysis: antithrombotic therapy, time to hospital consultation, tumor origin, quick-SOFA ≥ 2 on admission, SOFA on admission, TOKYO Grade III on admission, time to biliary decompression, mechanical ventilation, use of catecholamines, septic shock during stay, persistence of hematological failure after decompression, and persistence of renal failure after decompression.

## 3. Results

### 3.1. Study Population Characteristics

A total of 140 patients were included in the study. There were 88 female and 52 male patients. Median age was 61 years ± 17.88 (range 16–93 years), and 26% of patients were older than 75 years of age. Mean time between onset of symptoms and admission was 10 days ± 5.28 (range 1-21 days). The most common comorbidities in our population were history of biliary procedure (42%), cardiovascular disease (24%), diabetes (16%), hypertension (16%), stroke (6%), and antithrombotic treatment (11%). Acute cholangitis was lithiasic in most cases (69%), tumoral (15%), hydatic (13%), inflammatory (odditis), and iatrogenic (postcholecystectomy) in the other cases. Underlying causes for tumoral cholangitis were cholangiocarcinoma (7 patients), pancreas cancer (6 patients), vater ampulloma (4 patients), and gallbladder cancer (4 patients). All patients had ultrasound examination. Hepatobiliary ultrasound showed a biliary dilatation in 98.6% of the cases and evidence of etiology in 74% of the cases. Abdominal computed tomography (CT), bili MRI, and echoendoscopy were needed, respectively, in 25, 12, and 1 patients. Eighteen patients had cholangitis etiology diagnosed during endoscopic retrograde cholangiopancreatectomy (ERCP). Upon admission, 70% of patients presented with a systemic inflammatory response syndrome, and 41.4% of patients had a quick-SOFA score ≥ 2. Grades III, II, and I of TOKYO Grading severity were present, respectively, in 81%, 15%, and 4% of the cases. Mean SOFA score was 8 ± 3, 69 (range 0-16). Mechanical ventilation was required in 32 patients (23%) with mean duration of 3.42 ± 2.99 days (range 1–15 days). Catecholamines and renal replacement therapy were needed in, respectively, 31.4% and 6.42% of patients. Ceftriaxone + metronidazole were used as initial empirical antibiotherapy in 87%. Antibiogram based antibiotherapy was performed secondary in 13 patients. 92% of patients underwent biliary drainage. Mean time to biliary drainage was 1.58 ± 0.89 days (range 0–5 days). Endoscopic unblocking was the technique used in 76% of cases (106 patients), with sphincterotomy in most cases (94 patients). Surgical and percutaneous techniques were performed in, respectively, 22 and 3 patients. Mean duration of ICU stay was 6 ± 3.96 days (range 1-23 days). Overall, ICU mortality rate was 28%. 90% of these patients died due to cholangiosepsis.

### 3.2. Univariate and Multivariate Analysis of Risk Factors for Mortality in Severe Acute Cholangitis

Univariate analysis identified 19 variables that were significantly (*p* < 0.05) associated with mortality in severe acute cholangitis in our population (Table 3). The variables entered into the logistic regression model were antithrombotic therapy, time to hospital consultation (days), tumor origin, severity assessment upon admission (quick-SOFA ≥ 2, SOFA, TOKYO Grade III), organ support therapies during ICU stay (use of catecholamines, mechanical ventilation), persistence of renal failure after decompression, persistence of hematological failure after decompression, and septic shock during ICU stay. Variables considered but not retained in the final model were creatinine, total bilirubin, GCS, respiratory failure as they are included in the severity assessment scores upon admission, dialysis as it is highly correlated to persistence of renal failure after decompression, and admission from medical ward as we considered it is not clinically relevant. Multivariate analysis showed that mortality in our ICU population was significantly associated with the history of taking antithrombotic treatment (OR = 10.146), the time to hospital consultation (OR = 1.137), and the use of catecholamines (OR = 5.819) and mechanical ventilation (OR = 13.649) during ICU stay (Table 4).

## 4. Discussion

This is a single center retrospective study analyzing the factors related to mortality in patients with severe acute cholangitis in ICU. Four variables, including the history of taking antithrombotic treatment, the time to hospital consultation, and the use of catecholamines and mechanical ventilation during ICU stay, were associated with the mortality of ICU patients with severe acute cholangitis.

Since the first surgical biliary decompression attempt was only described in 1903, mortality associated with acute cholangitis treated without biliary drainage was close to 100%. Despite surgical advances and the introduction of antibiotics over the following decades, mortality remained at 50%. But it has decreased to 10-30% since 1980, with the development of endoscopic biliary drainage techniques and the various associated therapeutic modalities [1, 7, 8]. Current mortality rates vary between 9.6% and 37% [6, 9–11], and death is most often due to a multiorgan failure related to a refractory septic shock [1]. This is consistent with our results which showed a mortality rate of 28% and refractory septic shock as the leading cause of death.

There are few studies on the prognostic factors for severe acute cholangitis. Although the TG18/TG13 severity criteria are currently widespread and very precise for the diagnosis and assessing the severity, they are based on expert opinions and therefore requiring additional validation in clinical practice [12]. In Morocco, to our knowledge, there has been no study to investigate prognostic factors in critically ill patients with severe acute cholangitis. In this study, multivariate analysis identified four independent risk factors for mortality: history of taking antithrombotic treatment, time to hospital consultation, and the use of catecholamines and mechanical ventilation during ICU stay.  Table 5 summarizes the different prognostic factors found in different studies compared to those in our study.

Severity assessment scores such as TOKYO grading as well as the classic quick-SOFA and SOFA did not stand out as prognostic factors in our study. There are two possible reasons: (1) the inclusion of patients in the study was based on these scores and (2) admission to ICU is considered in itself as a prognostic factor [6]. On the other hand, use of catecholamines and mechanical ventilation (both reflects of hemodynamic and respiratory failures) did stand out as prognostic factors. Both criteria are included in the SOFA and the TOKYO grading. Furthermore, mechanical ventilation is also associated with its own complications. Organ failure is therefore the main prognostic factor and the detection of patients at risk of progression to organ failure by scores such as quick-SOFA, SOFA, or TOKYO would improve the prognosis. In a recent study [13], quick-SOFA was associated with high specificity but decreased sensitivity to predict severity (97% vs. 43%) and admission to intensive care (96% vs. 60%). However, it is an easily reproducible clinical tool in the emergency room. Particular attention is to be addressed to the elderly and the immune suppressed patients. Often these patients do not have clear and definite clinical symptoms to guide the diagnosis and are also more likely to deteriorate rapidly due to their limited physiological reserve.

In this study, mean time to hospital consultation was of 10 days. This delay in consultation, and therefore the delay in the administration of antibiotics and biliary drainage, will make infection control more difficult with poor treatment results, especially in patients with comorbidities [6, 14]. The use of traditional therapies (fire points, etc.) and difficult access to care in remote areas is still a real problem to be taken into account in local and national policies to raise awareness and provide care.

Early antibiotic therapy is as important as appropriate antibiotic therapy. Several studies have also reported inappropriate probabilistic antibiotic therapy as a prognostic factor [15], which underlines the importance of bile samples and blood cultures for secondary adaptation as well as knowledge of local ecology. Moreover, other study [10] has reported that the presence of ESBL organism was significantly associated with organ failure in bacteria cholangitis. The initial empiric antibiotherapy dose did not show significant association with mortality in this study, but further analysis based on bile sample results is needed. We did not study this etiological factor since the bile sample results were missing in some cases.

No other study, to our knowledge, has reported taking antithrombotics as a prognostic factor. A history of antithrombotic therapy may reflect the severity of the underlying comorbidity, can worsen or precipitate hematological failure, and may delay or complicate biliary drainage. This factor is included as prognosis factor in some ICU scores such as for trauma patients. Further studies are needed, but we should pay particular attention to these patients.

Emergency biliary decompression is the primary treatment for severe acute cholangitis. Increasing bile pressure promotes biliary sepsis dissemination and prevents biliary penetration of antibiotics. Antibiotic therapy, which main purpose is to limit the infection spread while waiting for biliary decompression, remains essential but is alone insufficient. Biliary drainage is then recommended in acute cholangitis regardless of severity, with the exception of a few nonserious cases progressing spontaneously under antibiotic therapy and initial resuscitation measures [16]. 92% of our patients underwent biliary decompression. Three patients died before, and nine patients' conditions improved while waiting for decompression and were transferred. Endoscopic drainage, less invasive and compatible with a better quality of life (no bile leak compared to external drainage), is the first choice technique despite the risk of pancreatitis post-ERCP [17]. In our study, endoscopic drainage was the golden standard. Surgical treatment was the first alternative if endoscopic treatment (hepatobiliary endoscopists and/or equipment) was unavailable. Percutaneous drainage, performed in 2% of cases, is a technique recently introduced to our center. Early biliary drainage is associated with less intrahospital mortality, 30-day mortality, and hospital costs, particularly within 48 hours of admission and regardless of severity [18, 19]. Procalcitonin has been proposed by some authors as a predictive indicator of severity and therefore of urgent biliary decompression [20], but this needs to be validated by more well-designed studies. According to previous studies, biliary drainage is the most important factors associated with mortality [9–11]. Time to biliary decompression did not stand out as a prognosis factor in our series. Our population study is different: only ICU patients with severe cholangitis are included. Plus, most of our patients (87%) underwent early biliary drainage, within 48 hours, associated with early aggressive resuscitation. Furthermore, Lee et al. [21] reported that drainage beyond 48 hours is associated with persistent organ failure and prolonged length of stay but does not significantly affect mortality.

RDW (*Red Cell Distribution Width*) is traditionally high in cases of ineffective erythropoiesis or excessive destruction of erythrocytes. The prognostic value of RDW in different acute or chronic inflammatory circumstances (cardiovascular, traumatic, neurological, septic…) and especially in critically patients is recently raised by several studies [22]. RDW is associated to admission to intensive care and bilirubin levels within a predictive mortality score [6] but needs to be validated by more studies.

Our results may not be generalized as they are limited by the retrospective design of our study and by the mono-centric recruitment, but this is the first large study of ICU patients with severe cholangitis identifying local prognostic factors. As with any other observational study, even after adjusting for clinically and statistically significant prognostic factors, other unmeasured factors may have contributed to patient mortality. All retrospective series on this question are limited by significant confounding factors. The key to a successful logistic regression model is to choose the correct variables to enter into the model. While it is tempting to include as many input variables as possible, this can dilute true associations and lead to large standard errors with wide and imprecise confidence intervals, or, conversely, identify spurious associations [23]. We first run the univariate analyses and then used only the variables meeting a preset cutoff for significance of *p* < 0.05 to run a multivariable model. We also considered the scientific plausibility and the clinical meaningfulness of the association while trying to avoid the use of highly correlated variables.

As multiple organ failure is often associated with mortality in severe acute cholangitis; predictive risk factors for organ failure should be further investigated. Finally, a local contextualized protocol emerged from this work and is being validated in practice. These procedures are provided in Supplementary Materials (available here).

## 5. Conclusions

History of taking antithrombotic treatment, the time to hospital consultation, and the use of catecholamines and mechanical ventilation during ICU stay were associated with the mortality of ICU patients with severe acute cholangitis in this study. Predictive risk factors for organ failure should be further investigated.

In addition, severe acute cholangitis management requires multidisciplinarity (anesthetists, intensivists, hepatobiliary endoscopists, surgeons, radiologists, and microbiologists) and implementation of standardized protocols.

## Figures and Tables

**Table 1:** TG18/TG13 diagnostic criteria for acute cholangitis. Adapted from Kiriyama et al. [24].

(A) Systemic inflammation (A-1) Fever (temperature > 38°C) and/or shaking chills (A-2) *Evidence of Inflammatory Response*. White blood cells (WBC) count < 4000 or >10000/mm^3^, C-reactive protein ≥ 10 mg/l.
(B) Cholestasis (B-1) *Jaundice*. Total bilirubin ≥ 20 mg/l. (B-2) *Abnormal Liver Function Tests*. AST, ALT, ALP, r-GTP (>1.5 × STD).
(C) Imaging (C-1) Biliary dilatation (C-2) Evidence of etiology on imaging (stricture, stone, stent, etc.)

Suspected diagnosis: one item in A + one item in either B or C. Definite diagnosis: One item in A + one item in B + one item in C.

Other factors may be helpful in diagnosis of acute cholangitis: right upper quadrant or upper abdominal pain, a history of biliary disease (gallstones, previous biliary procedures, biliary stent). In acute hepatitis, marked systematic inflammatory response is observed infrequently. Virological and serological tests are required when differential diagnosis is difficult.

ALP: alkaline phosphatase; r-GTP (GGT): r-glutamyltransferase; AST: aspartate aminotransferase; ALT: alanine aminotransferase; STD: upper limit of normal value.

**(a):** 

TG 18/TG13 severity assessment criteria for acute cholangitis. Adapted from Kiriyama et al. [24]
Grade III (severe): acute cholangitis + one dysfunction at least in any of the following systems: (i) *Cardiovascular*. Hypotension requiring vasopressors. (ii) *Neurological*. Disturbance of consciousness. (iii) *Respiratory*. PaO2/FiO2 ratio < 300 (arterial oxygen partial pressure to fractional inspired oxygen ratio). (iv) *Renal*. Oliguria, serum creatinine > 20 mg/l. (v) *Hepatic*. PT–INR > 1.5 (prothrombin-international normalized ratio). (vi) *Hematologic*. Platelet count < 100, 000/mm^3^. Grade II (moderate): acute cholangitis + any two of the following conditions: (i) Abnormal WBC count (>12000/mm^3^ or < 4000/mm^3^) (ii) Fever ≥ 39°C (iii) Age ≥ 75 years (iv) Hyperbilirubinemia (total bilirubin ≥ 50 mg/l) (v) Hypoalbuminemia (<0.7 × STD) Grade I (mild): acute cholangitis does not meet the criteria of “Grade III (severe)” or “Grade II (moderate)” acute cholangitis at initial diagnosis.

**(b):** 

Sequential Organ Failure Assessment (SOFA) score [25]. Based on the degree of dysfunction of 6 organ systems: respiratory, hematologic, hepatic, cardiovascular, neurological, and renal.					
Variables/score	0	1	2	3	4
PaO_2_/FiO_2_ (mmHg)	> 400	≤ 400	≤ 300	≤ 200	≤ 100
Platelets (×10^3^/mm^3^)	> 150	≤ 150	≤ 100	≤ 50	≤ 20
Bilirubin (mg/l)	< 12	12-19	20-59	60-119	> 120
Cardiovascular (*μ*g/kg/min)	No hypotension	MAP < 70 mmHg	Dopa ≤ 5 or Dobu (any dose)	Dopa > 5 or norepi ≤ 0.1	Dopa > 15 or norepi > 0.1
Glasgow coma scale	15	13-14	10-12	6-9	< 6
Creatinine (mg/l) or urine output	< 12	12-19	20-34	35–49 or < 500 ml/day	> 50 or < 200 ml/day

MAP: mean arterial pressure; Dopa: dopamine; Dobu: dobutamine; Norepi: norepinephrine.

**(c):** 

Quick-SOFA [26]	
Systolic blood pressure ≤ 100 mmHg	1 point
Respiratory rate ≥ 22 breaths/min	1 point
Glasgow coma scale ≤ 14	1 point

**Table 3:** Univariate analysis of risk factors for mortality in severe acute cholangitis.

Factors	Survivors (*N* = 101)	Nonsurvivors (*N* = 39)	*p* value	Brut OR (95% CI)
Age, mean ± SD	59.49 ± 18.96	64.36 ± 14.37	0.150	
Age ≥ 75 years, *n* (%)	27 (26.7%)	10 (25.6%)	0.896	0.94 (0.40-2.19)
Female gender, *n* (%)	67 (66.3%)	21 (53.8%)	0.170	
Comorbidities, *n* (%)				
Diabetes	15 (14.85%)	7 (17.94%)	0.414	
Cancer	3 (2.97%)	1 (2.56%)	0.690	
Heart disease	22 (21.78%)	11 (28.2%)	0.277	
Stroke	6 (5.9%)	2 (5.1%)	0.853	0.85 (0.16-4.43)
Antithrombotic therapy	5 (5%)	10 (25.6%)	≤0.001	6.62 (2.09-20.93)
History of complicated lithiasic disease	9 (8.9%)	2 (5.1%)	0.456	0.55 (0.11-2.68)
History of biliary procedure	41 (40.6%)	14 (35.9%)	0.610	0.82 (0.38-1.76)
Time to hospital consultation (days), mean ± SD	9.07 ± 5.011	12.03 ± 5.426	0.003	
Type of admission, *n* (%)				
Emergency	85 (84.2%)	27 (69.23%)	0.048	0.42 (0.17-1.00)
Surgical ward	15 (14.9%)	7 (17.9%)	0.652	1.25 (0.46-3.35)
Medical ward	1 (1%)	5 (12.82%)	0.002	14.70 (1.65-130.3)
Charcot triad, *n* (%)	94 (96.03%)	36 (92.3%)	0.563	
Etiology, *n* (%)				
Lithiasic	75 (74.3%)	22 (56.4%)	0.040	0.44 (0.20-0.97)
Tumor	10 (9.9%)	11 (28.2%)	0.007	
Hydatic	12 (11.9%)	6 (15.4%)	0.579	1.34 (0.46-3.88)
Severity assessment upon admission				
Septic shock, *n* (%)	7 (6.9%)	25 (64.1%)	≤0.001	
Respiratory failure, *n* (%)	11 (10.89%)	15 (38.5%)	≤0.001	
GCS, mean ± SD	14.52	13.07	≤0.001	
GCS< 15, *n* (%)	22 (21.8%)	25 (64.21%)	≤0.001	6.41 (2.86-14.37)
Quick-SOFA ≥ 2, *n* (%)	24 (23.76%)	27 (69.2%)	≤0.001	
SOFA, mean ± SD	6.5 ± 2.95	11.27 ± 3.066	≤0.001	
TOKYO Grade III, *n* (%)	76 (75.2%)	38 (97.4%)	0.002	
Laboratory finding upon admission, mean ± SD				
Creatinine, mg/l	23.20	37.18	0.001	
White blood cell count, /mm^3^	19718.4	23888.5	0.257	
C-reactive protein, mg/l	197.5	209.84	0.496	
Aspartate amino transferase, IU/l	166.91	208.25	0.185	
Alanine aminotransferase, IU/l	141.81	202.58	0.139	
Alkaline phosphatase, IU/l	364.92	478	0.052	
Total bilirubin, mg/l	106.5	174.76	0.002	
Prothrombin time < 50%, *n* (%)	33 (32.7%)	16 (41.02%)	0.353	
Organ support therapies				
Catecholamines, *n* (%)	21 (20.8%)	32 (82.1%)	≤0.001	17.41 (6.74-44.96)
Mechanical ventilation, *n* (%)	6 (5.9%)	26 (66.7%)	≤0.001	31.66 (10.96-91.41)
Dialysis, *n* (%)	2 (2%)	7 (17.9%)	0.001	10.82 (2.14-54.78)
Initial empiric antibiotherapy, *n* (%)				
Amoxicillin + clavulanic acid	13 (12.9%)	4 (10.3%)	0.671	0.77 (2.36-2.53)
Ceftriaxone + metronidazole	87 (86.1%)	35 (89.7%)	0.568	1.40 (0.43-4.57)
Time to biliary decompression (days), mean ± SD	1.39 ± 0.840	1.41 ± 1.186	0.910	
Decompression technique, *n* (%)				
Endoscopic	82 (81.2%)	29 (74.4%)	0.371	0.67 (0.28-1.61)
Surgical	16 (15.8%)	6 (15.4%)	0.947	0.96 (0.34-2.68)
Percutaneous	2 (2%)	1 (2.6%)	0.831	1.30 (0.11-14.78)
Persistence of renal failure after decompression, *n* (%)	3 (3%)	10 (25.6%)	≤0.001	11.26 (2.90-43.67)
Persistence of hematological failure after decompression, *n* (%)	5 (5%)	35 (89.7%)	≤0.001	168 (42.66-661.50)
ICU stay (days), mean ± SD	5.60 ± 3.65	5.62 ± 4.67	0.988	
Septic shock during ICU stay, *n* (%)	0 (0%)	35 (89.7%)	≤0.001	

**Table 4:** Multivariate analysis of risk factors for mortality in severe acute cholangitis.

Factors	*p* value	Adjusted odds ratio (95% confidence interval)
History of antithrombotic therapy	0.004	10.146 (2.125; 48.44)
Use of catecholamines	0.005	5.819 (1.71; 19.80)
Mechanical ventilation	≤0.001	13.649 (3.715; 50.148)
Time to hospital consultation	0.019	1.137 (1.023; 1.264)

**Table 5:** Prognostic factors in acute cholangitis in different studies.

Studies	Study design	Prognostic factors
Yıldız et al. [6] Turkey	Retrospective Suppurative acute cholangitis 104 ICU patients 2010-2015	(i) Total bilirubin ≥ 6.9 mg/dl (ii) RDW^1^ ≥ 14.45% (iii) Admission to ICU
Valsangiacomo et al. [9] Uruguay	Retrospective Suppurative acute cholangitis 81 patients 2002–2015	(i) Age > 65 years (ii) Male (iii) Septic shock on admission (iv) Time to biliary decompression
Ban Seok Lee et al. [10] South Korea	Retrospective Acute cholangitis 211 patients 2003-2011	(i) ESBL^2^ (ii) Total bilirubin (iii) Blood urea nitrogen (iv) Biliary decompression
Mohammed Aboelsoud et al. [11] USA	Retrospective Severe acute cholangitis 177 patients 2001–2012	(i) Albumin (ii) Total bilirubin (iii) SAPS-II (iv) Age (v) Time to biliary decompression
Gravito-Soares et al. [12] Portugal	Retrospective Acute cholangitis 183 patients 2017	(i) Systolic blood pressure < 90 mmHg (ii) Hypoalbuminemia (iii) Active neoplasia (iv) Tumor obstruction
Our study Fez, Morocco	Retrospective Severe acute cholangitis 140 patients 2009–2018	(i) Time to hospital consultation (ii) Catecholamines (iii) Mechanical ventilation (iv) History of antithrombotic therapy

^1^RDW: red cell distribution width. ^2^ESBL: extended-spectrum beta-lactamase.

## Data Availability

All data and tables used to support the findings of this study are included within the article.

## Supplementary Materials

Supplementary Materials The file includes our local contextualized procedure for the management of severe acute cholangitis and is provided both in its original language (French) and in English.
