# Supplementary material for: Factors Associated with Mortality in Severe Acute Cholangitis in a Moroccan Intensive Care Unit: A Retrospective Analysis of 140 Cases
Source: Gastroenterol Res Pract. 2021 Jan 27;2021:4583493. doi: 10.1155/2021/4583493 (PMC7861946; doi:10.1155/2021/4583493)
Supplement: Supplementary Materials — The file includes our local contextualized procedure for the management of severe acute cholangitis and is provided both in its original language (French) and in English. [file 4583493.f1.zip › Local protocol for the management of severe acute cholangitis (French Version).pdf]

Protocole simplifié de prise en charge des angiocholites aigues graves

L'organisation de la prise en charge, **multidisciplinaire**, doit se faire en étroite collaboration entre anesthésistes-réanimateurs, radiologues, endoscopistes, chirurgiens et microbiologistes selon un protocole codifié et adapté à chaque structure de soins.

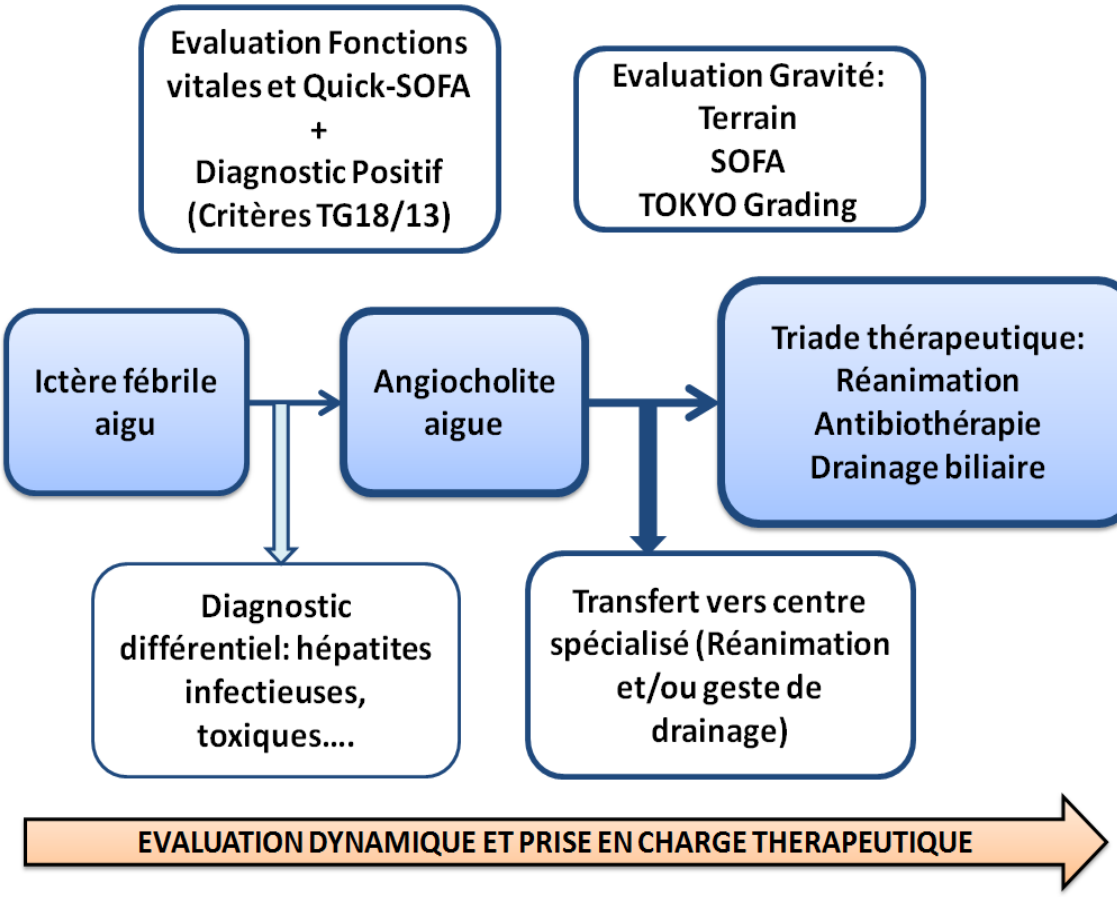

Organigramme de prise en charge de l'angiocholite aigue grave
(Adapté des Tokyo Guidelines 2018)

➔ **CONFIRMATION DIAGNOSTIQUE**

- ☐ Dès suspicion du diagnostic d'angiocholite aigu ➔ **Critères diagnostiques TG18/TG13**
- ☐ Si diagnostic définitif difficile, réévaluer le patient toutes les 6 à 12 h en utilisant les critères diagnostiques, pour le confirmer.

Critères diagnostiques de Tokyo (TG18/TG13) pour l'angiocholite aigue.

A. Inflammation systémique

A-1/ Fièvre (Température> 38°) **et / ou frissons.**

A-2/Réponse inflammatoire systémique : Leucocytes <4000 ou >10000 éléments/mm^3, CRP ≥ 10 mg/l.

B. Cholestase

B-1/ Clinique : Ictère et Bilirubine Totale ≥ 20 mg/l.

B-2/ Biologique : Bilan hépatique perturbé : ASAT, ALAT, PAL, GGT (> 1,5×N)

C. Imagerie

C-1/ Dilatation des voies biliaires.

C-2/ Evidence étiologique: obstacle visible (calcul, sténose, prothèse,…).

Diagnostic suspecté si: 1 critère A + 1 critère B ou C

Diagnostic confirmé si: 1 critère A + 1 critère B + 1 critère C

A-2 : Taux anormal de leucocytes, augmentation de la CRP ou d'autres indicateurs de l'inflammation.

B-2 : Augmentations du taux sérique des enzymes hépatiques (ASAT, ALAT, GGT, PAL)

D'autres facteurs peuvent contribuer au diagnostic de l'angiocholite aigue : colique hépatique, antécédent d'une pathologie biliaire (calculs biliaires, prothèse biliaire, intervention sur les voies biliaires).

L'hépatite aigue s'associe rarement à une réponse inflammatoire systémique marquée. Les sérologies virales sont recommandées en cas de diagnostic différentiel difficile.

C : **Echographie hépatobiliaire** en première intention.

TDM abdominale, Bili-IRM, échoendoscopie en deuxième intention.

➔ EVALUATION DE LA GRAVITE

☐ Avant confirmation diagnostique ➔ Evaluation clinique de la gravité : **quick-SOFA.**

**Hypotension artérielle (PAS ≤ 100 mmHg)**	**1 point**
**Fréquence respiratoire élevée (≥ 22/min)**	**1 point**
**Altération de conscience (GCS ≤ 14)**	**1 point**

☐ Si quick-SOFA ≥ 1 ➔ Admission en unité de soins intensifs + mise en condition :

- ✓ Monitorage standard : ECG, SpO2, pression artérielle non invasive.
- ✓ Oxygénothérapie.
- ✓ Position demi-assise si détresse respiratoire et/ou neurologique, lever de jambes si détresse hémodynamique.
- ✓ 2 voies veineuses périphériques.
- ✓ Antalgiques (Eviter AINS).
- ✓ Bilan biologique et réanimation hydro électrolytique initiale (Cristalloïdes +++)
- ✓ Réévaluation ± Transfert.

☐ Dès confirmation du diagnostic d'angiocholite aigu ➔ **Critères de gravité TG18/TG13.**

☐ Si angiocholite aigue grave Grade III et/ou terrain particulier ➔ Prise en charge en milieu de réanimation spécialisée.

☐ **Cinétique du score de SOFA** pendant le séjour en réanimation: au moment du diagnostic, dans les 24 h après le diagnostic puis toutes les 24 heures.

Critères de gravité de l'angiocholite aigue TG18/TG13

Grade III (Sévère) : Si angiocholite aigue + au moins 1 défaillance d'organes parmi les défaillances suivantes :

- ➔ **Cardiovasculaire:** Hypotension nécessitant le recours aux vasopresseurs.
- ➔ **Neurologique:** Trouble de conscience.
- ➔ **Respiratoire:** Détresse respiratoire aigue avec un rapport PaO_2/FiO_2 <300.
- ➔ **Rénale:** Oligurie, taux de créatinine >20 mg/l.
- ➔ **Hépatique:** TP-INR > 1.5.
- ➔ **Hématologique:** Taux de plaquettes <100,000/mm3.

Grade II (Modérée) : Si angiocholite aigue associée à 2 des critères suivants :

- ➔ Hyperleucocytose ou leucopénie (>12,000/mm^3 ou <4000/mm^3).
- ➔ Fièvre ≥ 39°C.
- ➔ Age > 75 ans.
- ➔ Hyper bilirubinémie (Taux de bilirubine totale > 50 mg/l).
- ➔ Hypo albuminémie (< 0.7 × N).

Grade I (Légère) : Si aucun des critères Grade III ni Grade II n'est présent au moment du diagnostic

Score de SOFA (Sequential Organ Failure Assessment)					
Variables/Score	0	1	2	3	4
PaO_2/FiO_2 (mmHg)	> 400	≤ 400	≤ 300	≤ 200	≤ 100
Plaquettes (×10^3/mm^3)	> 150	≤ 150	≤ 100	≤ 50	≤ 20
Bilirubine (mg/l)	< 12	12 - 19	20 - 59	60 - 119	> 120
Cardiovasculaire (µg/kg/min)	Pas hypotension	PAM < 70 mmHg	Dopa ≤ 5 or Dobu (any dose)	Dopa > 5 or nora ≤ 0,1	Dopa> 15 or nora > 0,1
Glasgow Coma Scale	15	13 - 14	10 - 12	6 - 9	< 6
Créatinine (mg/l) Ou diurèse	< 12	12 - 19	20 - 34	35 – 49 ou < 500 ml/24h	> 50 ou < 200 ml/24h
PAM : Pression artérielle moyenne, Dopa : Dopamine, Dobu: Dobutamine, Nora : Noradrénaline					

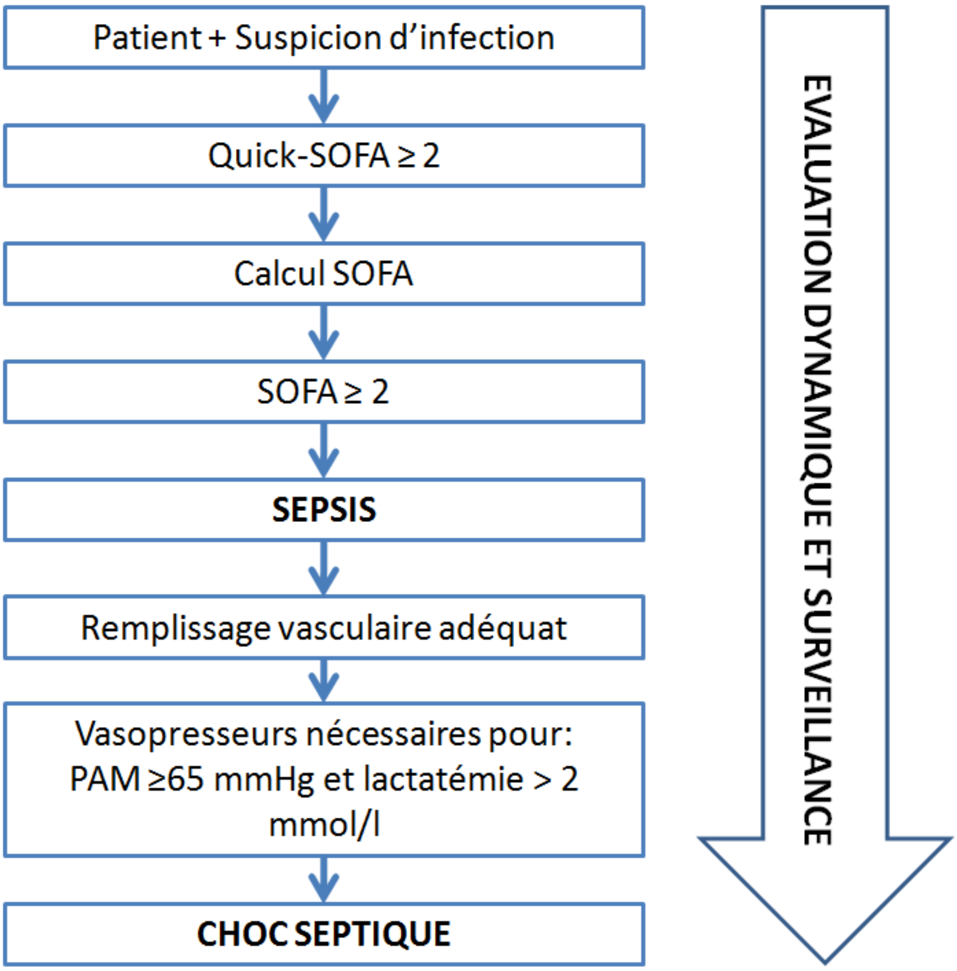

➔ **PRISE EN CHARGE THERAPEUTIQUE**

☐ Si réanimation spécialisée et/ou plateau technique pour drainage biliaire indisponible :

- ✓ Mise en condition initiale.
- ✓ Réanimation hydro électrolytique et démarrer antibiothérapie.
- ✓ Transfert régulé vers une structure spécialisée.

MESURES DE REANIMATION	
**Timing**	Dès suspicion du diagnostic
**Monitorage**	• Standard : ECG, SpO2, Pression non invasive, diurèse, température, glycémie capillaire, 2 VVP<br>• Avancé : Abord veineux central, pression artérielle invasive, PVC, débit cardiaque<br>• Biologique (personnalisé): NFS + plaquettes bilan hépatique, fonction rénale, bilan d'hémostase, bilan électrolytique et glycémie, CRP, groupage ABO-Rhésus, lactates, procalcitonine, gaz de sang artériel et veineux.
**Réanimation liquidienne**	• Cristalloïdes : Sérum salé 0,9%, Ringer lactate<br>• Commencer par 30ml/kg puis continuer si réponse hémodynamique positive et absence de surcharge pulmonaire.<br>• Colloïdes proscrits.
**Traitement vasopresseur ± inotrope**	• Noradrénaline et/ou adrénaline<br>• Objectif PAM ≥ 65 mmHg et lactates < 2 mmol/l<br>• Dobutamine si défaillance cardiaque à l'échocardiographie + signes clinico-biologiques de bas débit.
**PEC respiratoire**	• Oxygénothérapie<br>• Ventilation mécanique protectrice si nécessaire
**Traitement antalgique**	• Eviter AINS
**Traitements adjuvants**	• HSHC 200mg/j si choc réfractaire<br>• Insulinothérapie pour une glycémie < 1,8 g/L<br>• Produits sanguins, vitamine K et facteurs de coagulation<br>• PEC nutritionnelle<br>• Prophylaxie antithrombotique, de l'ulcère lié au stress<br>• Epuration extra rénale si indication rénale

<table>
<tr><th colspan="3">TRAITEMENT ANTIBIOTIQUE</th></tr>
<tr><td colspan="2">Timing</td><td>Dans l’heure</td></tr>
<tr><td colspan="2">Voie d’administration</td><td>Injectable intraveineuse</td></tr>
<tr><td rowspan="2">Molécules (Traitement empirique)</td><td>Angiocholite aiguë communautaire grave</td><td>Ceftriaxone + Métronidazole</td></tr>
<tr><td>Angiocholite aiguë grave associée aux soins / après CPRE</td><td>Pipéracilline/tazobactam
Ou
Ceftazidime + Métronidazole
Ou
Carbapénème (Imipénème ou Ertapénème)
±
Vancomycine si suspicion d’Entérocoque Faecium
(ou Tigécycline si allergie à la vancomycine)</td></tr>
<tr><td colspan="3">Antibiothérapie initialement empirique puis secondairement adaptée aux résultats des cultures et d’antibiogrammes (prélèvement de bile et/ou hémocultures).</td></tr>
<tr><td>Durée du traitement antibiotique</td><td colspan="2">✓ 4 à 7 jours si la source infectieuse est contrôlée (= drainage efficace et fonctionnel).
✓ 7 à 10 jours si angiocholite survenant sur prothèse biliaire et obstruction levée.
✓ 15 jours minimum si présence confirmée de Cocci Gram + type entérocoque ou streptocoque (vu le risque d’endocardite infectieuse).</td></tr>
</table>

DRAINAGE BILAIRE	
**Timing**	En urgence sans délai
**Méthode de drainage**	- Endoscopique transpapillaire par CPRE.<br>- Percutané échoguidé si échec ou indisponibilité de la CPRE.<br>- Chirurgical si échec ou indisponibilité des 2 premières.
**Méthode de drainage endoscopique**	Dépend de l'état du patient, de l'existence d'une coagulopathie et de la maitrise de l'endoscopiste.<br>± Drainage nasobilaire externe ou drainage interne par prothèse.<br>± Sphinctérotomie.<br>± Dilatation papillaire.
**Impératifs pour le geste**	- Information du patient et de sa famille.<br>- Monitorage standard minimal : ECG, SpO2, Pression artérielle non invasive, diurèse, 2 voies veineuses périphériques.<br>- Mesures de réanimation initiées (remplissage vasculaire, drogues vasoactives, transfusion, vitamine K, facteurs de coagulation …)<br>- Antibiothérapie administrée<br>- Anesthésie générale avec contrôle des voies aériennes pour les drainages endoscopique et chirurgical.<br>- Vigilance et communication avec l'opérateur (endoscopiste, radiologue interventionnel ou chirurgien).<br>- Prélèvement de bile per geste obligatoire.<br>- Surveillance des complications post geste : hémorragiques, septiques par décharges bactériennes ou perforation digestive, pancréatite post CPRE…
