# Supplementary material for: Factors Associated with Mortality in Severe Acute Cholangitis in a Moroccan Intensive Care Unit: A Retrospective Analysis of 140 Cases
Source: Gastroenterol Res Pract. 2021 Jan 27;2021:4583493. doi: 10.1155/2021/4583493 (PMC7861946; doi:10.1155/2021/4583493)
Supplement: Supplementary Materials — The file includes our local contextualized procedure for the management of severe acute cholangitis and is provided both in its original language (French) and in English. [file 4583493.f1.zip › Simplified local protocol for the management of severe acute cholangitis (English Version).docx]

## Simplified local protocol for the management of severe acute cholangitis (English Version)

Severe acute cholangitis management requires multidisciplinarity (anesthetists, intensivists, hepatobiliary endoscopists, surgeons, radiologists and microbiologists) and implementation of standardized and customized local procedures.


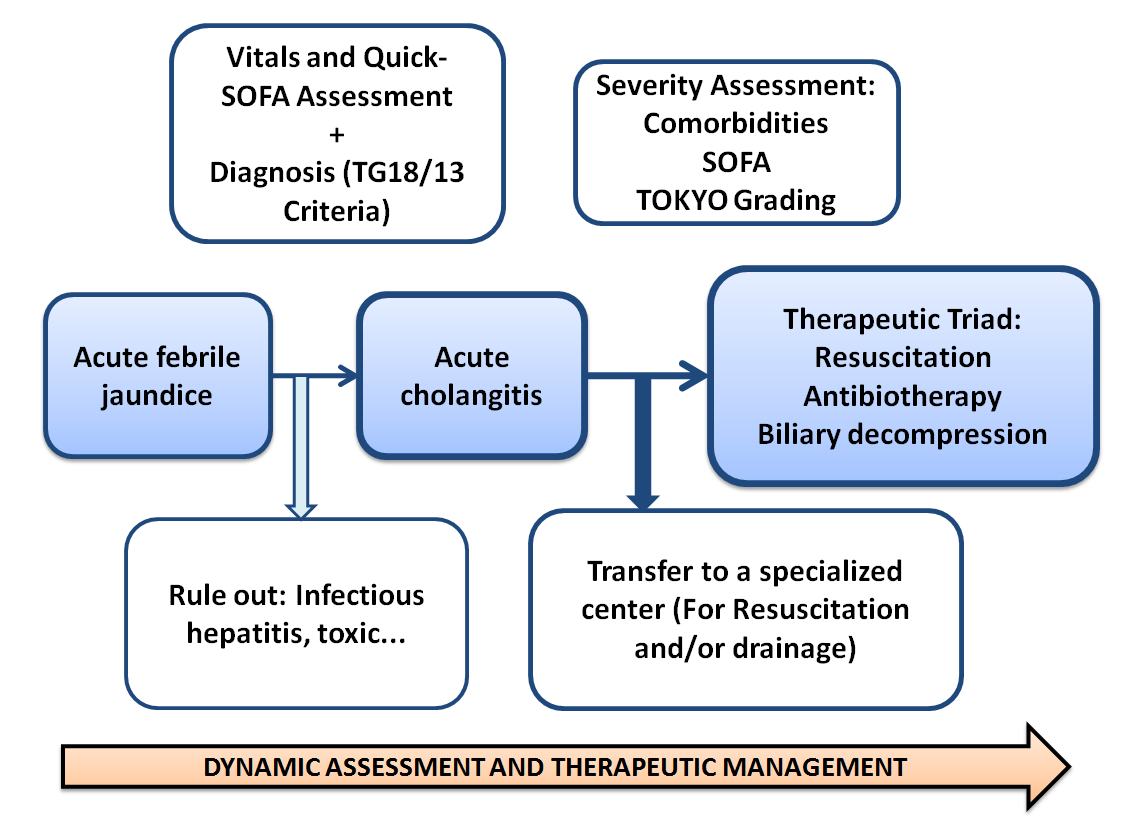


Flowchart for the management of severe acute cholangitis

(Adapted from Tokyo Guidelines 2018)

- **DIAGNOSIS CONFIRMATION**
- Once the diagnosis of acute cholangitis is suspected 🡺 **TG18/TG13 Diagnostic criteria for acute cholangitis.**
- If definitive diagnosis is unclear, reassess the patient every 6 to 12 hours following diagnostic criteria to confirm.

**TG18/TG13 Diagnostic criteria for acute cholangitis**

| A Systemic inflammation  A-1/ Fever (Temperature > 38°C) and/or shaking chills.  A-2/Evidence of inflammatory response: White Blood Cells (WBC) count < 4000 or >10000/mm^3^, C-Reactive Protein ≥ 10 mg/l.  B. Cholestasis  B-1/ Jaundice: Total Bilirubin ≥ 20 mg/l.  B-2/ Abnormal liver function tests: AST, ALT, ALP, r-GTP (> 1.5×STD).  C. Imaging  C-1/ Biliary dilatation.  C-2/ Evidence of etiology on imaging (stricture, stone, stent, etc.). |
| --- |
| Suspected diagnosis: one item in A + one item in either B or C.  Definite diagnosis : one item in A + one item in B + one item in C. |
| A-2: Abnormal white blood cell count, increased CRP or other indicators of inflammation.  B-2 : Increased serum liver enzymes (ASAT, ALAT, GGT, PAL)  Other factors may be helpful in diagnosis of acute cholangitis : Right upper quadrant or upper abdominal pain, a history of biliary disease (gallstones, previous biliary procedures, biliary stent).  In acute hepatitis, marked systematic inflammatory response is observed infrequently. Virological and serological tests are required when differential diagnosis is difficult.  C: Hepatobiliary ultrasound as first-line test.  Abdominal CT, Bili-MRI and echo-endoscopy as second-line tests. |

ALP Alkaline phosphatase, r-GTP (GGT) r-glutamyltransferase, AST aspartate aminotransferase, ALT alanine aminotransferase,

- **SEVERITY ASSESSMENT**
- Before confirming diagnosis 🡺 Clinical assessment of severity: **quick-SOFA.**

| **Systolic Blood Pressure ≤ 100 mmHg** | **1 point** |
| --- | --- |
| **Respiratory Rate ≥ 22 breaths/min** | **1 point** |
| **Glasgow Coma Scale ≤ 14** | **1 point** |

- If **quick-SOFA ≥ 1** 🡺 **Admission** to Intensive Care Unit **AND** First Management :
- Standard monitoring: Electrocardiogram (Heart rate and rhythm), oxygen saturation, non invasive blood pressure, temperature, urinary output.
- Oxygen therapy.
- Half-seated position, head at 45° if respiratory and/or neurological distress, raise legs if hemodynamic distress.
- Pain relievers (Avoid non-steroidal anti-inflammatory drugs).
- Laboratory assessment: blood count + platelets, liver function, kidney function, haemostasis, electrolytes, glycaemia, C - reactive protein, arterial blood gases and lactates, procalcitonin. Blood typing.
- Fluid resuscitation (Cristalloïds +++)
- Re-assesment ± Transfer if specialized resuscitation and/or biliary drainage non available.
- Once diagnosis of acute cholangitis is confirmed 🡺 **TG18/TG13 Severity Criteria.**
- If Grade III severe cholangitis and/or specific co morbidities 🡺 Management in a specialized resuscitation setting.
- **SOFA score Assessment during ICU stay:** at the time of diagnosis, within 24 hours after diagnosis and then every 24 hours.

| TG 18/TG13 SEVERITY ASSESSMENT CRITERIA FOR ACUTE CHOLANGITIS |
| --- |
| Grade III (Severe) : Acute cholangitis + one dysfunction at least in any of the following systems :   - Cardiovascular: Hypotension requiring vasopressors. - Neurological: Disturbance of consciousness. - Respiratory: PaO2/FiO2 ratio < 300 (arterial oxygen partial pressure to fractional inspired oxygen ratio). - Renal: Oliguria, serum creatinine > 20 mg/l. - Hepatic: PT – INR > 1.5. (Prothrombin - International Normalized Ratio). - Hematologic: Platelet count < 100,000/mm3.   Grade II (Moderate) : Acute cholangitis + any two of the following conditions:   - Abnormal WBC count (>12000/mm3 or <4000/mm3). - Fever ≥ 39°C. - Age ≥ 75 years. - Hyper bilirubinemia (Total bilirubin ≥ 50 mg/l). - Hypo albuminemia (< 0.7 × STD).   Grade I (Mild): acute cholangitis does not meet the criteria of ‘Grade III (severe)’ or ‘Grade II (moderate)’ acute cholangitis at initial diagnosis |

| SEQUENTIAL ORGAN FAILURE ASSESSMENT (SOFA) SCORE | | | | | |
| --- | --- | --- | --- | --- | --- |
| Variables/Score | 0 | 1 | 2 | 3 | 4 |
| PaO_2_/FiO_2_ (mmHg) | > 400 | ≤ 400 | ≤ 300 | ≤ 200 | ≤ 100 |
| Platelets (×10^3^/mm^3^) | > 150 | ≤ 150 | ≤ 100 | ≤ 50 | ≤ 20 |
| Bilirubin (mg/l) | < 12 | 12 - 19 | 20 - 59 | 60 - 119 | > 120 |
| Cardiovascular (μg/kg/min) | No hypotension | MAP < 70 mmHg | Dopa ≤ 5 or Dobu (any dose) | Dopa > 5 or norepi ≤ 0,1 | Dopa> 15 or norepi > 0,1 |
| Glasgow Coma Scale | 15 | 13 - 14 | 10 - 12 | 6 - 9 | < 6 |
| Creatinine (mg/l)  or urine output | < 12 | 12 - 19 | 20 - 34 | 35 – 49  or < 500 ml/day | > 50  or < 200 ml/day |
| MAP : Mean Arterial Pressure, Dopa :Dopamine, Dobu: Dobutamine, Norepi : Norepinephrine | | | | | |

**
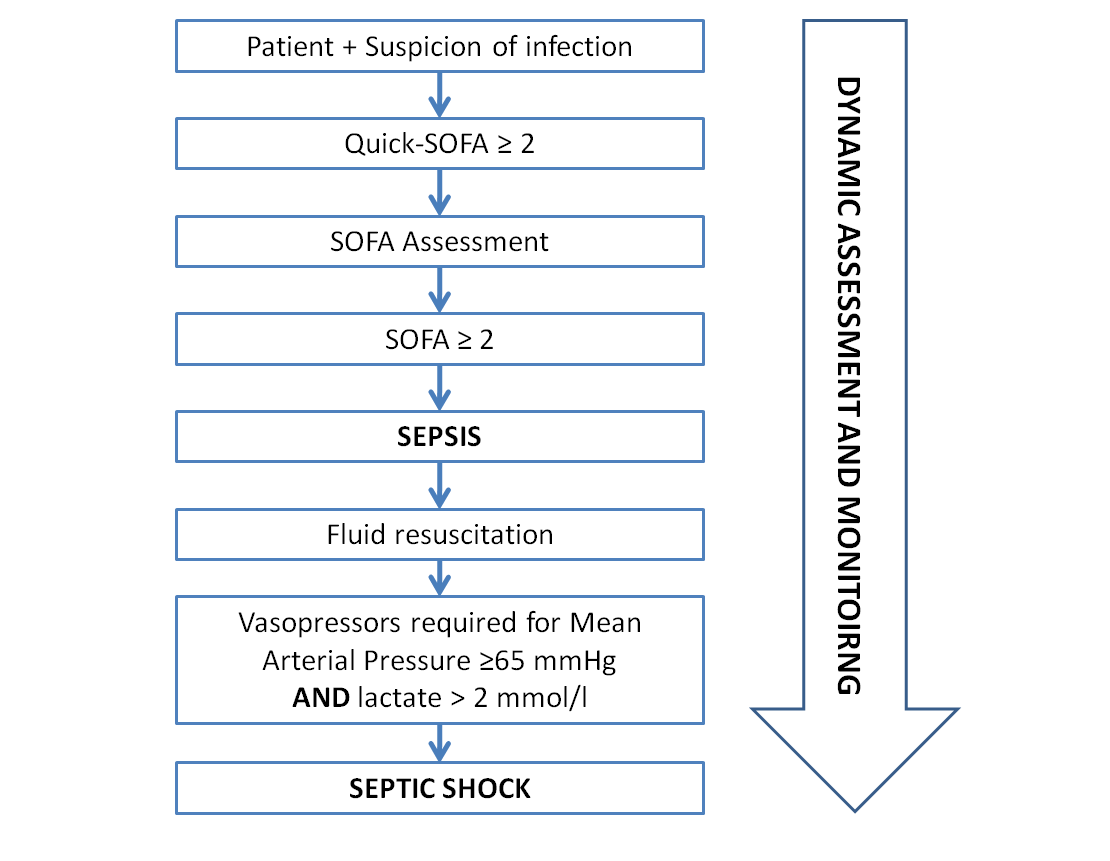
**

- **THERAPEUTIC MANAGEMENT**
- If specialized resuscitation and/or technical platform for biliary drainage not available:
- First management and conditionning.
- Fluid resuscitation AND Initiate antibiotic therapy.
- Regulated transfer (Through Emergency Call Service) to a specialized facility.

| **RESUSCITATION MEASURES** | |
| --- | --- |
| **TIMING** | As soon as the diagnosis is suspected |
| **MONITORING** | - Standard: heart rate and rhythm, blood pressure, oxygen saturation, temperature, urinary output, glycemia. - 2 peripheral venous lines 18 - 16 Gauge ± central venous line. - Advanced: central venous line. Invasive blood pressure, Central venous pressure, cardiac output. - Laboratory (customized): blood count + platelets, liver function, kidney function, haemostasis, electrolytes, glycaemia, C- reactive protein, arterial and venous blood gases and lactates, procalcitonin. Blood typing. |
| **FLUID RESUSCITATION AND RENAL SUPPORT** | - Cristalloids: Saline 0.9%, Lactate Ringer - Start by 30ml/kg and continue if positive hemodynamic response and no pulmonary overload. - Colloïdes PROSCRIBED. - AVOID NEPHROTOXICS. - Dialysis if indication. |
| **VASOPRESSORS**  **± INOTROPIC THERAPY** | - Noradrénaline et/ou adrénaline - Objectif PAM ≥ 65 mmHg et lactates < 2 mmol/l - Dobutamine si défaillance cardiaque à l’échocardiographie + signes clinico-biologiques de bas débit. |
| **RESPIRATORY SUPPORT** | - Oxygen therapy. - Tracheal intubation and mechanical ventilation if required. |
| **ANALGESIC THERAPY** | - Avoid non-steroidal anti-inflammatory drugs. - Use morphine with caution. |
| **OTHER ORGAN SUPPORT THERAPIES** | - Hydrocortisone 200mg/d ay if refractory shock. - Insulin therapy to maintain blood glucose < 1.8 g/L - Blood products, vitamin K and clotting factors if required - Nutrition - Thromboembolic prophylaxis and stress ulcer prevention. |

| **ANTIBIOTIC THERAPY** | | |
| --- | --- | --- |
| **TIMING** | | Within one hour |
| **ADMINISTRATION** | | Intravenous injection |
| **EMPIRICAL THERAPY** | **Community-acquired severe acute cholangitis** | Ceftriaxone + Metronidazole |
|  | **Care-associated severe acute cholangitis**  **or**  **after** **endoscopic retrograde cholangio-pancreatectomy (ERCP)** | Piperacillin/tazobactam  **Or**  Ceftazidime **+** Metronidazole  **Ou**  Carbapenem (Imipenem or Ertapenem)  **±**  Vancomycin if suspicion of Enterococcus Faecium  ( or Tigecycline if allergy to vancomycin ) |
| Antibiotherapy **initially empirical** then **secondarily adapted** to the results of cultures and antibiograms (**bile sampling** and/or blood cultures). | | |
| **DURATION OF ANTIBIOTIC THERAPY** | - **4 to 7 days** if the infectious source is controlled (= efficient and functional drainage). - **7 to 10 days**  if cholangitis develops on a biliary prosthesis and obstruction is resolved - **15 days minimum** if the presence of Cocci Gram + such as Enterococcus or streptococcus is confirmed (given the risk of infectious endocarditis). | |

| **BILIARY DRAINAGE** | |
| --- | --- |
| **TIMING** | - Urgently without delay |
| **DRAINAGE METHOD** | - Transpapillary endoscopy by ERCP. - Percutaneous echo-guided if ERCP fails or is not available. - Surgical if failure or non availability of the first 2. |
| **ENDOSCOPIC DRAINAGE METHOD** | Depends on the patient's condition, the existence of coagulopathy and the endoscopist expertise.  ± External nasobilary drainage or prosthesis.  ± Sphincterotomy.  ± Papillary dilatation. |
| **IMPERATIVES FOR THE PROCEDURE** | - Patient / Family information and consent. - Minimal standard monitoring: Electrocardiogram, oxygen saturation, non-invasive blood pressure, urinary output, 2 peripheral venous lines. - Resuscitation measures initiated (vascular filling, vasoactive drugs, transfusion, vitamin K, coagulation factors ...). - Antibiotherapy administered - General anesthesia with airway control for endoscopic and surgical drainage. - Vigilance and communication with the operator (endoscopist, interventional radiologist or surgeon). - Bile sampling is mandatory. - Monitoring for post-procedure complications: haemorrhagic, bacterial sepsis, digestive perforation, post ERCP pancreatitis... |
